# Cancer Survivorship in the Age of YouTube and Social Media: A Narrative Analysis

**DOI:** 10.2196/jmir.1569

**Published:** 2011-01-17

**Authors:** Wen-Ying Sylvia Chou, Yvonne Hunt, Anna Folkers, Erik Augustson

**Affiliations:** ^3^New College of FloridaSarasota, FLUnited States; ^2^Tobacco Control Research BranchNational Cancer InstituteNational Institutes of HealthBethesda, MDUnited States; ^1^Health Communication and Informatics Research BranchNational Cancer InstituteNational Institutes of HealthBethesda, MDUnited States

**Keywords:** narrative communication, cancer survivors, social media, qualitative research, linguistics, health communication.

## Abstract

**Background:**

As evidenced by the increasing popularity of YouTube (www.youtube.com), personal narratives shared through social media are an area of rapid development in communication among cancer survivors. Identifying the thematic and linguistic characteristics of YouTube cancer stories can provide a better understanding of this naturally occurring communication channel and inform social media communication efforts aiming to use personal stories to reach individuals with serious illnesses.

**Objective:**

The objective of our study was to provide an in-depth description of authentic personal cancer stories. Through a linguistically based narrative analysis of YouTube stories, the analysis explicates the common attributes of these narratives.

**Methods:**

Informed by narrative theories, we conducted an iterative, bottom-up analysis of 35 YouTube videos identified by the search terms “cancer survivor” and “cancer stories”. A list of shared thematic and linguistic characteristics was identified and analyzed.

**Results:**

A subnarrative on the cancer diagnosis was present in 86% (30/35) of the stories under analysis. These diagnostic narratives were characterized by dramatic tension, emotional engagement, markers of the loss of agency or control, depersonalized reference to the medical personnel, and the unexpectedness of a cancer diagnosis. The analysis highlights the themes of story authenticity and emotional engagement in this online communication medium.

**Conclusions:**

Internet advances have enabled new and efficient exchange of personal stories, including the sharing of personal cancer experience among cancer survivors and their caregivers. The analytic results of this descriptive study point to the common characteristics of authentic cancer survivorship stories online. Furthermore, the results of this descriptive study may inform development of narrative-based communication, particularly in maintaining authenticity and emotional engagement.

## Introduction

A growing body of research points to the importance of storytelling as a cancer communication tool. Through various storytelling contexts, including support groups, patient testimonials, medical encounters, and communication interventions, personal cancer stories have been shown to have a positive health impact for listeners and storytellers alike [1-4]. Survivors’ stories have been used as a vehicle for modeling coping skills, providing social and emotional support, and sharing information and resources [5]. Recently, cancer communication efforts have begun to adapt narratives as a tool for changing health behavior. For example, Kreuter and colleagues have demonstrated that effective use of survivors’ narratives increased mammography uptake among African-American women [1,5]. One explanation of narrative’s effectiveness is that survivors’ direct experience with cancer makes them credible and effective messengers of information conveyed through their personal stories. In this way, narrative communication is seen as offering unique advantages over traditional expository or didactic communication in the context of promoting desirable health behaviors.

Data suggest that individuals with chronic deceases are increasingly using online media to engage in health-related social networking, provide mutual support, and share stories [6,7]. As such, the Internet is an important venue for sharing personal stories about cancer. Paralleling the increased use of social media among cancer survivors, a growing number of cancer control organizations are using patient narratives as a communication device, for a variety of intents and purposes. For example, in its “Survivor Interviews” series, The Lance Armstrong Foundation (ie, LiveStrong) features 200 videotaped cancer narratives of men, women, and caregivers and invites people to watch the survivors’ stories “to learn about cancer, to deal with a diagnosis and to hear firsthand about their experiences.” The American Cancer Society’s “Stories of Hope” series offers a repository of videotaped survivors’ stories across different cancer topics, intended to provide “inspiration, hope, and support”. The American Legacy Foundation’s Legacy for Health “Letters” campaign features farewell letters of four women battling terminal tobacco-related cancers, with the goal of raising public awareness of the dangers of smoking and encouraging people to quit. Many cancer treatment centers also use cancer narratives in their marketing and communications efforts. The Mayo Clinic’s “Patient Stories” catalogues videotaped survivors’ stories as a window “into our institution, to our model of care, and how the Mayo approach to medicine can change people’s lives.”

The use of cancer narratives to raise public awareness, provide information and support, and change behavior for people living with cancer has clear instrumental value. However, communication science has not kept pace with the rapid uptake of this new cancer communication medium. Moreover, new-media research can benefit greatly from multidisciplinary approaches, including “a mixture of quantitative and qualitative methodologies appropriate for the specific problem under investigation” [8]. In order to better understand storytelling in new media, such as YouTube, added value can be drawn from qualitative, in-depth descriptive research examining authentic social media discourse to explicate particular attributes and functions of cancer narratives.

Linguistic analysis provides the tools to understand both the form and the function of narrative. Though linguistic methods have long been used to examine other types of illness stories (eg, those of war survivors, psychiatric patients, prisoners), this method has never been applied to cancer narratives [9-11]. A linguistically based narrative analysis can contribute to this inquiry in two ways. First, based on existing theories of narrative, a linguistic approach explicates the storytelling process, going beyond content analysis (*what* is said) to describe the storytelling process (*how* it is said) and identify common characteristics of cancer stories. Moreover, this iterative, bottom-up approach provides a micro-level analytic method to uncover key elements of authentic cancer narratives to yield a better understanding of these stories.

In terms of locating a Web source to conduct social media linguistic analysis, the Internet website YouTube (www.youtube.com) presented an ideal venue, due to its proliferation of user-generated cancer stories. A free video-sharing site created in 2005, YouTube has over 100 million videos, many of which contain personal stories about health and illnesses. With its high accessibility and wealth of user-generated content, the site provides a natural environment in which to conduct an in-depth examination of authentic, patient-generated cancer narratives. 

The aims of our analysis were twofold: (1) to identify key characteristics of the naturally occurring survivors’ video stories, including linguistic features shared across stories, and (2) to examine the functions of these attributes in the storytelling medium. To address these research aims, our research team conducted a linguistic analysis of 35 YouTube stories posted by cancer survivors, with the primary focus on the posters’ verbal construction of the cancer diagnosis experience. The study exemplifies a new and innovative approach to describe online narrative communication. By stepping outside traditional communication research methods and using insights from linguistic research, we are able to obtain insights on the form and function of the narrative attributes in cancer survivors’ stories. The results have the potential of informing future research and practice using social media and personal narrative for cancer communication efforts.

## Methods

Data for analysis were extracted from the YouTube site (Multimedia Appendix 1). With more than 100 billion views per day, a significant amount of health- and cancer-related content is being shared on the site [12]. We conducted two consecutive rounds of narrative analysis of the English-language YouTube site in October 2008 and January 2009 using the search terms “cancer survivor” and “cancer stories.” The research team excluded videos with the primary purpose of entertainment, advertisement, news broadcast, public service announcement, live speech, and artistic expression or those that were highly scripted and elicited by someone other than the survivor. 

During round 1 of the data analysis, the top 20 clips rated by the YouTube ranking algorithm as most relevant were extracted. We recorded the selected videos’ title, URL, length, number of views and viewer rating (1-5 stars, with 5 being the best) on the day of extraction, author of the video, and the affiliation of the author. All clips were transcribed in their entirety. The research team then analyzed the selected videos to generate hypotheses and to inform the development of a codebook. This open-ended analysis was guided by research aim #1, namely, the identification of key characteristics of survivors’ stories. More specifically, the characteristics under analysis included thematic (content-level) as well as discursive (linguistic-level) features. The team of analysts set out to identify and agree on a number of common themes and linguistic features in the data set.

To inform this iterative analytic process, we familiarized ourselves with the narrative analysis literature, particularly the seminal work on narrative syntax by sociolinguist William Labov [13]. The framework posits that naturally occurring personal-experience narratives generally follow a shared *narrative syntax,* consisting of a set of structural elements, including an *abstract*, *orientation*, *complicating action*, *evaluation*, *result/resolution*, and *coda*, each marked by specific linguistic properties. For example, temporally ordered clauses characterize the complication action section of the narrative, moving the events being narrated forward. Based on the narrative syntax, the three coders identified the segments of the first 20 stories and took extensive notes on their direct observations.

After the first round of coding, intercoder reliability was ascertained by having the three coders each code the same set of transcripts to reconcile differences and reach consensus. The project’s qualitative framework prompted the use of group consensus building over statistical tests to ensure reliability. 

Round 1 provided a hypothesis-generating method and, based on research team discussions, we decided to focus round 2 analysis particularly on the diagnostic narratives. We constructed a coding sheet, using 15 additional YouTube videos identified by the same search criteria. The goal of round 2 was to validate and more accurately describe the observations and hypotheses laid out in round 1. All coded results were entered into ATLAS.ti version 6 qualitative software (ATLAS.ti Scientific Software Development GmbH, Cologne, Germany) for ease of counting and excerpt identification.

## Results

### Prevalence of Diagnostic Narratives

The analysis revealed that the majority of YouTube posters begin their stories with an abstract (“It’s time that I tell my story of surviving cancer”), immediately followed by a set of orientation clauses. Uniformly throughout the data set, this orientation or setup involves recounting the event of finding out a cancer diagnosis. Hereafter, we term these narratives of cancer diagnosis as “diagnostic narratives.” The subsequent analysis will describe thematic and linguistic attributes of these diagnostic narratives. 

As shown in Table 1, 86% of all stories from rounds 1 and 2 consisted of a distinct segment of a diagnostic narrative. 

We found that diagnostic narratives were generally set up with a sense of normalcy, portraying life before the diagnosis as ordinary. They were also found to contain a number of specific linguistic features, including explicit orientations to specific time and space, prevalence of direct reported speech, use of the generic pronoun “you,” and depersonalized reference to the medical personnel. Excerpt 1 presents an example of diagnostic narrative, where an ovarian cancer survivor is recounting a seemingly ordinary day with a friend at the mall, when she first suspected that something was wrong.

Excerpt 1

One day, my friend J and I we were at the mall, and I had one of those pains and she said what is that, and I said I don’t know, and she said well you should really go see your doctor, and I said oh, womanly cramps, she said …

In this segment, the poster frames the event of diagnosis through orientation clauses, followed by a series of verbal exchange (direct reported speech) between her friend and herself, through which the suggestion of checking it with a doctor is raised and she initially dismisses it. Notice the description suggests an initial lack of suspicion about cancer and a sense of normalcy, implied by the ordinary nature of “womanly cramps”. 

Excerpt 1 illustrates common thematic and linguistic elements of cancer diagnostic narratives. They can be summarized as fulfilling four narrative functions: the unexpectedness of a cancer diagnosis, dramatic tension with a high level of emotional engagement, absence of control, and finally, the depersonalized reference of medical personnel. Table 2 lists the narrative functions and the linguistic characteristics serving these functions. The last column shows a sample of frequency of occurrence for several of the features coded. Except for temporal/spatial orientation, the linguistic features are counted only in round 2 (n = 14) for purposes of validating the observations made in round 1.

**Table 1:** Prevalence of diagnostic narratives (DNs) in YouTube survivorship stories

Analysis	Total number of stories	Stories with DNs (% prevalence)
Round 1	20	16 (80%)
Round 2	15	14 (93%)
Rounds 1 and 2 combined	35	30 (86%)

**Table 2:** Common discursive elements in diagnostic narratives (DNs)

Narrative functions	Representative linguistic features	Frequency of occurrence of selected linguistic features (% of DNs)
Framing of cancer as unexpected and diagnosis as unforgettable	Temporal and spatial orientation clauses	Temporal/spatial orientation, n = 25/30 (83%)
	Prefacing diagnosis with a sense of normalcy	
Dramatic tension and emotional engagement	Direct reported speech/thought	Direct reported speech, n = 10/14 (71%)
	Evaluative languages (eg, negation and emphatic adverbials)	
Marked absence of control	Use of generic pronoun “you”	Generic “you,” n = 4/14 (29%)
	Passive voicing	
	Nonagentive verbs	
Depersonalized reference to the medical personnel	Use of “they”	n = 13/14 (93%)
	Unnamed/unspecified referent	
	Passive voicing	

### Cancer as Unexpected and the Moment of Diagnosis as Unforgettable

The first feature of the diagnostic narratives is the presence of orientation framing, typically marked by temporal orientation and description of a sense of normalcy. In other words, these narratives frequently contain explicit mention of time (calendar time such as “November 13, 2004” or time relative to the narrator’s life, such as “the day before my 30th birthday”), space, and memorable life events surrounding the event of receiving a cancer diagnosis. As seen in Excerpts 2 and 3, this orientation framing is hypothesized to create dramatic tension leading up to diagnosis. It also functions to emphasize the speaker’s own vulnerability, as well as the seemingly randomness and the disruptive power of cancer.

Excerpt 2

For me life basically consists of basketball, football, soccer, and video games. But in May of 2006 I was diagnosed with cancer in my left arm.”

Excerpt 3

In 2004, things in my life were going great. I looked great, I felt great, and there was never even a hint, never even a whisper that there may be a problem.

In Excerpt 2, the narrator describes his precancer identities through a list of activities and hobbies. This life was abruptly interrupted—at a specific time (“May of 2006”) by the cancer diagnosis. Similarly, in Excerpt 3, the narrator emphasizes the “great” life she had prior to cancer. The description of normalcy is marked with positive descriptor, repetitions on the adjective “great,” the use of the emphatic adverbials “never even,” and the words “hint” and “whisper,” all implicating the unexpected nature of a cancer diagnosis.

Such explicit temporal and spatial orientation to the event of diagnosis occurred in 83% of the 30 stories (see Table 2). While some merely mention the time, others add evaluations to the receipt of a diagnosis. Excerpt 4 illustrates the poster’s evaluation of the event of finding out that he has cancer.

Excerpt 4

Um, January 22nd, that was the day, the worst day of my life when I found out that I had stage four lung cancer of all things. Um, that’s, just wipes you out! Whole family, everybody.You think you’re gonna die.

The prevalence of the mention of specific time and place of diagnosis and the explicit and often negative comment (eg, “the worst day of my life”) suggests the saliency and unforgettable nature of the event of receiving a cancer diagnosis in the speakers’ construction of cancer stories. 

### Creation of Dramatic Tension and Emotional Engagement

The YouTube cancer diagnosis stories share a sense of dramatic tension and high emotional engagement. Such sentiment is best illustrated through the frequent use of direct reported speech or thought (n = 10/14, 71%). Also termed “constructed dialogue” by Tannen to reflect the “constructed” nature of such expressions (as opposed to actual verbatim repetition), direct reported speech or thought represents a speaker’s use of voices from a past event during storytelling [14]. Narrative researchers have commonly associated the use of direct reported speech with the creation of a dramatic tension and sense of immediacy to the event being narrated [15-17]. For instance, in Excerpt 5 at the beginning of this diagnostic narrative, the poster describes a conversation with her doctor, where suspicion of breast cancer was raised. Segments containing reported speech are marked in bold type.

Excerpt 5

Um, but I said, **I think I felt something in the shower. He said, Well you’re young, I’m sure it’s nothing, but let me check it out anyway.**

The use of direct reported speech highlights the immediacy of the interaction between the narrator and her doctor, pulling the audience into the event leading up to learning about a cancer diagnosis. The poster uses the doctor’s words to convey the lack of suspicion of breast cancer, echoing the unexpected nature of the diagnosis. In Excerpt 6, we again see the news of a cancer diagnosis narrated through direct reported speech. This time, the speaker animates the doctor’s voice to give the cancer diagnosis.

Excerpt 6

We scheduled a core biopsy and after the biopsy he came back and said, **you have cancer**.

In Excerpt 6, the bad news is described through a reported speech animating the doctor’s voice. As it appears in the video, the direct and blunt style of the statement not only conveys the shock and emotional distress associated with the diagnosis, but also suggests a perceived lack of support from the doctor in navigating the medical world. Both Excerpts 5 and 6 show how direct reported speech helps the posters provide vivid descriptions of interactions between the them (as patients) and their providers, friends, and family members at the time of diagnosis. Serving to temporally move the story forward, this series of back-and-forth exchanges can also be seen as creating a dramatic tension in the narrative, engaging the audience into the plot being narrated.

Similar to reported speech, direct reported thought was also used by posters to recount the time of diagnosis: 

Excerpt 7

I think the biggest question that ran through my mind was, **how could this be happening to me?**

The question (“how could this be happening to me?”) represents the narrator’s direct reported thought, as is seen within the video by the distinct pause and the shift in intonation immediately prior to this statement. The reported internal monologue suggests his emotional engagement in the storytelling. 

In addition to the use of direct reported speech and thought, the analysis revealed a high prevalence of evaluation clauses in the diagnostic narratives. Narrative researchers generally agree on the crucial role of evaluation in storytelling. In fact, evaluative language (operationally defined as clauses that reflect the teller’s personal point of value) has been identified as the essential element that turns a series of recounted *events* into a *story* [13,18,19]. In illness narratives, evaluative language helps create a dramatic tension in storytelling. In addition to explicit indication of the tellers’ stance toward the events being narrated (known as *external evaluations),* there are a large number of “syntactic, lexical, and phonological mechanisms embedded within the clauses” to indicate the teller’s perspective (known as *internal evaluations*) [19]. These external and internal evaluations can be observed linguistically. For example, in Excerpt 8, a poster comments on her reactions to the cancer diagnosis through a number of linguistic features.

Excerpt 8

I couldn’t believe that this was happening to me. I have three young, small children. Things were going perfect in my life.

Marked by negation (“I couldn’t...”), perception verbs (“believe”), and emphatic descriptors (“perfect”), this excerpt contains highly evaluative language, contributing to the heightened dramatic tension and emotional engagement during the diagnostic narrative. 

### Absence of Control

A third narrative function observed in diagnostic narratives is the posters’ absence of control. Used interchangeably with the word “agency,” control is a theme that has been examined in illness narratives in psychology, linguistics, and anthropology [20-22]. The concept of control (ie, verbal positioning of self as being in control) has been analyzed in a wide range of health discourses, including patient-provider communication, illness stories, and health literacy assessment, and has been linked to coping and the construction of illness identity [22]. In recounting the events leading up to a cancer diagnosis, YouTube posters signal the lack of control through their stories. Linguistic evidence of such lack of control includes the use of passive voicing (“*I was operated on*”), nonagentive expressions (“*They gave me three months to live*”; “*I was diagnosed with lung cancer*”), and, most notably, the switch from the first-person pronoun “I” to the generic second-person pronoun “you.”

Consistent with prior research on pronouns in illness narratives, use of the generic pronoun “you” is found in statements where the poster signals a strong lack of control and negative affect [23]. In Excerpt 9, an ovarian cancer survivor uses “you” when describing the experience of being in a “surreal place” upon receiving the news of a cancer diagnosis. The second-person generic pronoun is marked in bold type.

Excerpt 9

You find yourself just in a surreal place like **this can’t really be happening to me, it was a mistake**.

This statement marks a shift from a narrative dominated by the first-first pronoun “I” to the generic pronoun “you,” accompanied by direct reported thought of disbelief at the point where she has lost a sense of control. The pronoun switch can be interpreted as fulfilling two possible functions. The first, consistent with Brown and Gilligan, indicates distance from the narrated event [24]. In both Excerpts 9 and 10, the narrators signal detachment from the news of cancer and death. Secondly, the pronoun shift moves the story from the immediate narrated event to an evaluation of psychological and emotional responses to diagnosis. In Excerpt 10, the shift happens when the narrator juxtaposes a description of coping (“breaking it down”) with her feeling of being overwhelmed:

Excerpt 10

I’m doing pretty good today and I’m breaking it down. But at the time **you**’re overwhelmed by this news. It’s just too much! **You** think **you**’re dying you know, according to them. And the way **you** cough, I felt like it.

The narrator comments on her receipt of a cancer diagnosis with external evaluation, “It’s just too much”. Similar to what’s found in Excerpt 4 (“...just wipes you out”), this “I” to “you” switch generalizes the reaction to everyone, and may also signal a sense of helplessness, especially the face of one’s mortality. In describing her cough, “you” is used to signal one’s loss of control to the physical symptoms. Note the sarcasm hinted in the phrase “according to them:” the narrator displays a contrasting attitude toward the diagnosis from the medical professionals. The portrayal of medical professionals is discussed n the next section.

Finally, related to the observation that the generic “you” is used to signal loss of control, this pronoun is frequently used when talking about death and dying. This co-occurrence is illustrated in Excerpt 11:

Excerpt 11

I remember one night when I was lying awake at the hospital...shaking, sweating, and not knowing why. But then realizing that **you**’re so close to death that **you** don’t know what to do.

Note the shift from “I” to “you”, when the narrator switches from recounting her physical experience of suffering from severe symptoms in the hospital to describing her mental state and a sense of confusion and disorientation facing mortality. This use of “you” in describing one’s being “so close to death” and not knowing what to do any further signals the lack of control common in the diagnostic narratives.

### Depersonalized Reference to Medical Personnel

In the video narratives, posters generally adopted a neutral or antagonistic stance toward the medical staff (primarily oncologists and surgeons). Regardless of stance, medical personnel were referenced in a highly depersonalized manner, often referred to simply as “they” or “the doctor.” The depersonalized reference suggests the tangential role they play in the diagnostic narratives. It further reinforces the survivors’ ownership of the cancer experience. 

Excerpt 12

When I went to see the doctor, they told me it was nothing, they told me it was a fluid-filled cyst and not to worry about it.

The use of “the doctor” and “they” in Excerpt 12 is typical of the way doctors were portrayed in the stories. In fact, except in videos affiliated with particular organizations (eg, hospitals), medical personnel were rarely given any prominence in the data set. Even when they were described to perform or say something significant, they were not mentioned by name, as illustrated in Excerpt 13:

Excerpt 13

Six weeks before my 40th birthday, I was diagnosed with testicular cancer. About three days after that I was on the operating table and the surgeon removed my left testicle.

While the specific temporal orientation (including the speaker’s age at the time of diagnosis and number of days between the news and the surgery) is commonly found throughout the data set, the speaker’s lack of personal evaluations here is unusual. However, the lack of emotional evaluation is juxtaposed with deliberate directness and terseness. The speaker describes the surgery following the diagnosis with a matter-of-fact tone of voice. Note that except in this excerpt, his surgeon was not once mentioned throughout his entire video, making the linguistic choice of “the surgeon” highly marked in this context. 

In conclusion, the analysis found that in survivor-generated YouTube stories, medical personnel play an insignificant role and are depersonalized when they are referenced. In some cases, the speakers expressed negative emotions or disagreements toward them (such as in Excerpt 10), while in other cases they merely served to complete the narrated event without being given any prominence (such as in Excerpts 12 and 13). 

## Discussion 

Personal narratives hold enormous potential as cancer communication tools, especially as social media continue to transform the way people interact with cancer-related information. However, the particular attributes or functions that make a cancer narrative effective as a communication tool are still not well understood. This information is critically important to cancer control organizations and other developers of cancer messages who hope to use storytelling as a vehicle for raising public awareness about cancer risk, providing information and support to cancer patients, and changing attitudes and behavior. The current study was undertaken to better understand the common linguistic elements of cancer narratives (eg, form) and the functions of these elements in the narratives’ ability to reach and engage audiences.

Survivors’ stories shared a common narrative syntax, characterized by a set of *orientation* statements describing the experience of being diagnosed with cancer, a series of *complicating actions* describing the events following the initial diagnosis, a variety of evaluation statements attempting to make meaning out of the cancer diagnosis, and, finally, a result or resolution to the diagnosis event. Understanding the syntax of naturally occurring cancer stories is useful, to the extent that narrative communication is most effective when it is perceived as authentic and credible. Cancer communication programs wishing to create narrative content that will resonate with audiences would do well to model their narrative syntax on what is observed here.

Social media approaches to cancer communication that include the use of personal narratives would further benefit from understanding the specific linguistic components that make a cancer narrative effective. Within the overall narrative structure described above, a number of shared linguistic themes and features were identified, which appear to serve important storytelling functions. Orientation statements frequently contained explicit orientations to space and time that conveyed a sense of normalcy prior to the diagnosis. As a storytelling device, this juxtaposition between “before” and “after” helped to establish the cancer diagnosis as unexpected, and the moment of diagnosis as unforgettable. The narratives were also characterized by frequent use of direct reported speech and highly evaluative language. From a storytelling perspective, these linguistic features served to build dramatic tension, increase realism, and induce emotional responses. Because the success of cancer communication efforts depends largely on creating emotional engagement with message content, there is value in understanding how specific linguistic features can be leveraged to create content that will form an emotional connection with the audience. 

The information gleaned from the linguistic analysis also provides health communicators, practitioners, and researchers with a window into the cancer diagnosis experience, from the patient point of view. Many of the identified key linguistic attributes reflected patients’ own perspectives on the experience with cancer, and this knowledge may shed light on the story posters: as they expressed it in their own words, the YouTube posters generally placed heavy emphasis on the moment of diagnosis, and positioned the news as unexpected and themselves as helpless. Evidence of this lack of agency was observed in the linguistic analysis as the use of passive voicing, nonagentive expressions, and use of the generic second-person pronoun “you.” However, over time they moved to construct a coherent account of being a cancer survivor and taking control of their lives, regardless of the prognosis. In contrast, medical staff were found to be infrequently mentioned and depersonalized. One can surmise that the posters, in comparison with other cancer survivors, may display several unique traits; namely, they tended to adjust to the cancer diagnosis and many assumed an activist/advocate role in cancer survivorship. 

These insights into survivors’ personal perspectives, including common challenges and the process toward better adjustment, have implications in the context of patient counseling. Echoing narrative medicine, which refers to clinical practice fortified by narrative competence, namely, “the capacity to recognize, absorb, metabolize, interpret, and be moved by stories of illness,” clinicians working with cancer patients may better understand patients’ perspectives and offer better support by listening to their stories [25].

Finally, from a methodological perspective, the analysis illustrates the utility of qualitative linguistic analysis to uncover key elements of cancer narratives: our approach presents to the health communication field a new and innovative analytic method that can be adopted for other types of qualitative health research using data such as from open-ended interviews, focus groups, or patient support groups. In this way, the linguistic analysis of the storytelling process can complement traditional content analysis.

### Study Limitations

This study has several limitations. The first is related to sample size. The nature of this in-depth qualitative analysis permitted the use of only a small selection of YouTube videos, therefore making any quantitative analysis uninformative. However, the results of the current descriptive analysis can potentially inform future research on large databases or corpora. Using natural-language processing and computational techniques, a similar type of narrative analysis can be done through a semiautomated coding scheme informed by the study results. For instance, key linguistic features such as reported speech or pronoun shifts can be automatically coded in a large set of survivors’ videos, and the results may be correlated with characteristics of illness experience, including prognosis, coping ability, and thoughts about and mention of one’s mortality. Adding these quantitative components to a future narrative study can confirm and substantiate existing qualitative observations.

The second limitation has to do with the low generalizability of the YouTube data across the population of cancer survivors. Racial and socioeconomic status disparities in online survivor narratives have been documented; in particular, stories by minorities are underrepresented on the Internet, including on YouTube [26]. The current study confirmed this observation: only 2 of the 35 video stories were posted by survivors of non-European decent. The fact that upper-middle-class Americans of European decent are more likely to post YouTube stories suggests limited generalizability of current results across the population. With more YouTube and other social media data, we will be able to better understand the variation in cancer narratives across racial, ethnic, and cultural groups. Moreover, YouTube posters, as compared with other cancer survivors, tend to be open to sharing personal experience in public and attempting to portray a coherent story, as well as having a more optimistic vision about cancer. 

Finally, on a more fine-grained level, we acknowledge that the analysis has excluded some aspects of language, in particular, prosodic features including intonation and pitch. Narrative research has found that prosody has important narrative functions, especially in accentuating the evaluative components of storytelling [27]. For this study, in an effort to focus on readily “codeable” features for the purpose of informing future analyses and designing narrative-based interventions, we left prosody out of the analysis. 

### Conclusions

This study presents a novel, linguistically oriented approach to analyzing the form and function of patient narratives situated in the discourse of social media. Such an analysis provides a better understanding of how Youtube posters use language to construct the illness experience and specifically the cancer diagnosis through this interactive online medium. The findings on the common attributes shared among Youtube cancer stories have the potential to inform future health communication efforts aiming to use personal narratives and social media. 

## Multimedia Appendix 1

Link list of YouTube videos under analysis
