# Supplementary material for: Cancer Survivorship in the Age of YouTube and Social Media: A Narrative Analysis
Source: J Med Internet Res. 2011 Jan 17;13(1):e7. doi: 10.2196/jmir.1569 (PMC3221357; doi:10.2196/jmir.1569)
Supplement: Supplementary file 1 [file jmir_v13i1e7_app1.pdf]

Link	Title	Length (minutes)	Date Posted	Notes
http://www.youtube.com/watch?v=xI16WQwwsE8	Thyroid Cancer Story	4:08	24/11/2008	
http://www.youtube.com/watch?v=nyZPlRHwf4k	Cancer Story	6:37	22/10/2008	
http://www.youtube.com/watch?v=HvuURupZoMw	Hodgkins Lymphoma Cancer Entry One - finding peace - My Story	7:33	21/02/2009	
http://www.youtube.com/watch?v=MXh5GfaCATs	Hodgkins Lymphoma Cancer Entry Two - treatment begins - My Story	8:57	28/02/2009	
http://www.youtube.com/watch?v=zzXvsO9fyWk	Hodgkins Lymphoma Cancer Entry Three - the routine - My Story	5:26	12/03/2009	
http://www.youtube.com/watch?v=DuiG-OQVnJs	Hodgkins Lymphoma Cancer Entry Four - my trust - My Story	6:08	16/03/2009	
http://www.youtube.com/watch?v=pwvF_Y_9iX8&feature=related	Breast Cancer Surviver: Rose Gerber	6:29	15/10/2007	
http://www.youtube.com/watch?v=xijpVbAyHHE	My Cancer survival story			Removed by user
http://www.youtube.com/watch?v=TRINsh7zJnY	Diagnosed With Cancer While Pregnant	5:56	23/05/2008	
http://www.youtube.com/watch?v=rBPPmUDEdZg	Brenda Ladun Story	5:01	26/01/2008	
http://www.youtube.com/watch?v=9LclPbmUT_g	Rebekah Gibbs on cancer	5:24	17/04/2008	
http://www.youtube.com/watch?v=8l8zDj89RfY	Team Medicine Stories - Bill and Prostate Cancer	3:03	23/03/2009	
http://www.youtube.com/watch?v=yodfW8PJJKc	You Can Thrive! Breast Cancer Survivor Stories	5:33	30/09/2008	
http://www.youtube.com/watch?v=-ynrcwNd0BA	Augusta Williams' Story of Breast Cancer Survival	8:50	07/11/2008	
http://www.youtube.com/watch?v=rlWnjla8toM	Broken Hope - A Young Adult Breast Cancer Story	2:43	21/04/2009	
http://www.youtube.com/watch?v=j9Pe7W-yO2g	My Cancer and Chemotherapy Story	8:24	08/08/2007	
http://www.youtube.com/watch?v=8YLbjZIdmwM	My cancer story	3:51	11/09/2008	
http://www.youtube.com/watch?v=3W9F4ftqZe0	Jessica's Story	3:54	21/04/2009	
http://www.youtube.com/watch?v=j28PPEt8KG8	Amazing Story	1:00	01/06/2007	
http://www.youtube.com/watch?v=R6sNbgMmUS0	Charron Walker, Breast Cancer Survivor & Founder of the Young Survivors' Network on			Removed by user
http://www.youtube.com/watch?v=8DkaLrVnLHI	Cancer Survivor Book	9:48	25/02/2009	
http://www.youtube.com/watch?v=HkhHYZ0b94U	Lisa, breast cancer survivor, helps others with cancer			Removed by user
http://www.youtube.com/watch?v=OFjS2mAcOoI&feature=related	You Don't Have To Die When Your Doctor says	7:57	13/05/2009	
http://www.youtube.com/watch?v=P3fXU_9gTjg	My testicular Cancer Story	1:40	30/01/2008	
http://www.youtube.com/watch?v=SdJoke3x_zU	My cancer story by Eric Larson	9:44	14/03/2007	
http://www.youtube.com/watch?v=3t9p-vuTG80	My cancer story	4:46	14/04/2007	
http://www.youtube.com/watch?v=-29tzyFACQg	Coralee's Breast cancer story	6:45	18/02/2007	
http://www.youtube.com/watch?v=kwWqEsw7caU	My cancer story	2:56	08/02/2007	
http://www.youtube.com/watch?v=RabWbJRaxes	Cancer survivor’s miraculous story Part 1	10:43	08/07/2007	
Part 2: http://www.youtube.com/watch?v=VKw_6bViV3U	Cancer Survivor's Miraculous Story Par 2	3:18	08/07/2007	
http://www.youtube.com/watch?v=SbPPS5cXK1c	Cancer survivor	7:33	07/09/2008	
http://www.youtube.com/watch?v=qiJLmglw6Pc	Breast Cancer Survivor Story: In Her Own Words	4:03	04/09/2007	
http://www.youtube.com/watch?v=I4ZqhTopO4g	Rosemary's Cancer Survival Story	8:43	05/08/2008	
http://www.youtube.com/watch?v=uKUQO6M_wTk&feature=related	Beating breast cancer during business school: Katie's Story	2:22	01/05/2008	
